# Mining Druggable Sites in Influenza A Hemagglutinin: Binding of the Pinanamine-Based Inhibitor M090

**DOI:** 10.1021/acsmedchemlett.4c00502

**Published:** 2024-11-28

**Authors:** Aitor Valdivia, Maria Rocha, F. Javier Luque

**Affiliations:** † Departament de Nutrició, Ciències de l’Alimentació i Gastronomia, Facultat de Farmàcia i Ciències de l′Alimentació - Campus Torribera, Universitat de Barcelona, Prat de la Riba 171, 08921 Santa Coloma de Gramenet, Spain; # Doctorate in Biotechnology, Departament de Nutrició, Ciències de l’Alimentació i Gastronomia, Facultat de Farmàcia i Ciències de l′Alimentació - Campus Torribera, Universitat de Barcelona, Prat de la Riba 171, 08921 Santa Coloma de Gramenet, Spain; ‡ Institut de Biomedicina (IBUB), Universitat de Barcelona, 08028 Barcelona, Spain; § Department of Life Sciences, University of Coímbra, Calçada Martim de Freitas, 3000-456 Coímbra, Portugal; ∥ Institut de Química Teòrica i Computacional (IQTCUB), Universitat de Barcelona, 08028 Barcelona, Spain

**Keywords:** Influenza A, Hemagglutinin, Ligand binding, Inhibition, Molecular dynamics, Free energy calculations

## Abstract

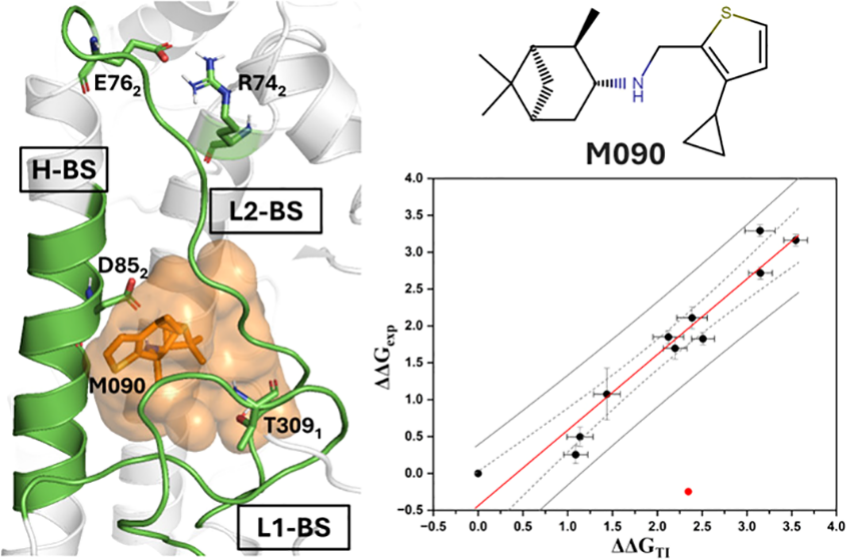

Assessing the binding mode of drug-like compounds is key in structure-based drug design. However, this may be challenged by factors such as the structural flexibility of the target protein. In this case, state-of-the-art computational methods can be valuable to explore the linkages between structural and pharmacological data. Following this strategy, extended molecular dynamics simulations and thermodynamic integration calculations are used to examine the binding of the potent antiviral inhibitor M090 and related pinanamine-based analogues, covering a 250-fold difference in inhibitory potency to the influenza A hemagglutinin, which is essential for virus entry and membrane fusion. This analysis has disclosed the hydrophobic shielding effect played by the 3-cyclopropylthiophene moiety in M090. Furthermore, the results support the negative effect of the resistance-induced E74_2_ → D mutation, which should weaken the binding by increasing the structural flexibility of the L2-BS loop. The results pave the way to exploration of the antiviral activity of novel compounds.

Mapping druggable pockets in therapeutically relevant proteins is a fundamental step in structure-based drug design. They should encode geometrical and physicochemical features suitable to accommodate drug-like compounds,^1,2^ including hot spots that can make major contributions to the binding affinity. Moreover, a proper balance between regions of high stability and high flexibility is also required.^3−7^ However, the structural plasticity of targets such as the influenza A hemagglutinin (HA), which is crucial for viral infectivity,^8−13^ may severely limit the success of drug discovery. However, the notion of HA as a druggable target has gained increasing support due to the discovery of small molecules that interfere with the role played in virus entry and membrane fusion.^14^

HA promotes entry of the virus into the cell by recognizing the sialic acid (SA) residues attached to cell membrane receptors. On the other hand, in the interior of the endosome, a large-scale conformational rearrangement of HA is triggered upon acidification, leading to the release of the fusion peptide (FP), which is hidden in the stem. The pH-induced structural remodeling promotes the fusion of viral and host membranes, enabling the release of the viral genetic material.^15,16^

From a structural point of view, HA is a homotrimer located on the viral envelope. Each protomer is expressed as a single, inactive precursor known as HA_0_, and maturation to the active form requires proteolytic cleavage by host cell proteases, leading to chains HA_1_ and HA_2_ (Figure 1).^17^ Two structural domains can be distinguished in HA: the globular head, which contains the receptor binding domain that mediates binding to the host cell membrane, and the stem region, which maintains the whole structure bound to the viral membrane. Three main strategies have been explored in the search of small molecules targeting HA:^18,19^ (1) altering the HA maturation via inhibition of host proteases required for proteolytic cleavage or alternatively through blockage of HA glycosylation;^20^ (2) impairing the recognition of the host receptor, resorting, for instance, to SA-mimic peptides;^21^ and (3) preventing the fusion between viral and host cell membranes by impeding the dynamical changes that trigger the release of the FP.^22−24^

**Figure 1:**
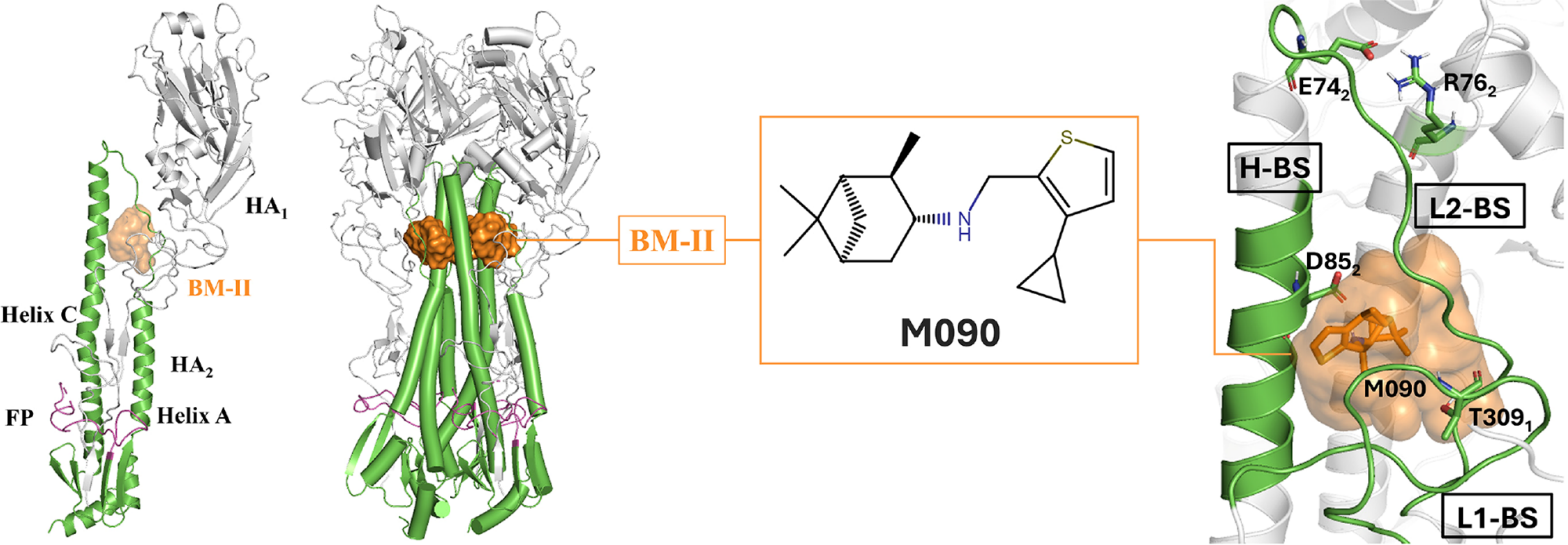
Structural representation of HA. The monomeric chains HA_1_ (gray) and HA_2_ (green) formed after proteolytic cleavage assemble, forming a trimer where the fusion peptide (FP) (magenta) remains buried until acidification in the endosome. The putative binding site of M090 (BM-II), which is shaped by an α-helix (H-BS) and two loops (L1-BS and L2-BS), is displayed as an orange surface that encloses M090 (shown as sticks).

Inspection of the available X-ray structures reveals that fusion inhibitors bind to two sites in the stem region. One corresponds to the pocket occupied by *tert*-butylhydroquinone (TBHQ) (PDB IDs 3EYK and 3EYM)^25^ and arbidol (PDB IDs 5T6N and 5T6S),^26^ consisting of residues located at helices A and C from protomer 1 and C′ from protomer 2. The other is the groove filled by JNJ4796 (PDB IDs 6CFG and 6CF7),^27^ (*S*)-F0045 (PDB ID 6WCR),^28^ and CBS1117 (PDB ID 6VMZ).^29^ This information is valuable not only to explore the structure–activity relationships but also to assess the putative binding mode of novel compounds.

Given the structural complexity of HA and its conformational plasticity, the occurrence of novel druggable sites cannot be ruled out. Indeed, other sites have been suggested for a diverse set of compounds, such as *N*-benzyl-4,4-disubstituted piperidines^30^ and pinanamine-based antivirals.^31,32^ The most potent inhibitor of this latter series of compounds, M090 (Figure 1), showed an EC_50_ of 0.3 μM against H1N1 A/Guangzhou/GIRD/07/2009 virus-infected MDCK cells, EC_50_ values of 0.34 and 0.1 μM in a plaque reduction assay against H1N1 A/California/07/2009 and A/Texas/04/2009, respectively, and EC_50_ values of 1.5–6.9 μM in cytopathic assays against several strains (H1N1, H3N2, H7N3, and H9N2). Moreover, M090 exerts its inhibitory activity in the early steps of the virus life cycle by targeting HA, and resistance selection experiments revealed the occurrence of the E74_2_ → D mutation in HA_2_. Finally, molecular modeling studies reported by Zhao et al.^32^ suggested that M090 binds at the interface defined by the α-helix L80_2_–A96_2_ (H-BS) and by loops P301_1_–K318_1_ and M59_2_–L73_2_ (L1-BS and L2-BS, respectively) (Figure 1). In this putative binding mode, the thiophene ring forms contacts with I308_1_, Y316_1_, and W92_2_, the pinane moiety fills the region shaped by A65_2_ and V66_2_, and the cyclopropyl ring interacts with F88_2_ and P307_1_. In addition, the N–H group forms a hydrogen bond (HB) to T309_1_.

In the absence of experimental structural evidence, this work aims to assess the proposed binding mode of M090 by synergistically combining the structural information on HA with computational studies—atomistic MD simulations (Table 1) and free energy calculations—and the pharmacological information gained from antiviral assays for M090-related compounds. The results are examined to gain insight into the effect of chemical changes on the inhibition potency and the impact of resistant-associated mutations.

**Table 1:** Summary of the Simulated Systems Used in This Work^a^

system	binding mode	code	starting structure	simulated time	ligand release
apo, wild type	–	Awt	homology model	2 μs	–
apo, E74→D mutant	–	Amut	snapshot at 1 μs of Awt	2 μs	–
holo, wild type	BM-I	H1wt-I	homology model	1 μs	no
		H2wt-I	snapshot at 1 μs of Awt	1 μs	1 out of 3
	BM-II	H1wt-II	homology model	2 μs	no
		H2wt-II	snapshot at 1 μs of Awt	1 μs	no
		H3wt-II	homology model	1 μs	no
		H4wt-II	snapshot at 2 μs of Awt	1 μs	no
holo, E74→D mutant	BM-II	H1mut-II	homology model	1 μs	no
		H2mut-II	snapshot at 2 μs of Awt	1 μs	no
holo, wild type	BM-R	H1wt-R	homology model	1 μs	1 out of 3
		H2wt-R	snapshot at 1 μs of Awt	0.5 μs	1 out of 3

a The representations of the three binding modes (BM-I, BM-II, and BM-R) are shown in Figure 2A1, Figure 2B1, and Figure 2C1, respectively.

To assess the structural stability of the binding mode proposed by Zhao et al. (denoted BM-I, Figure 2A1),^32^ a complex formed by HA (H1N1 A/Virginia/ATCC3/2009) and three M090 molecules (stoichiometric ratio 1:3), each bound to one of the three sites in the trimer, was built up. This strategy was also adopted in previous studies,^33^ thus enabling a check of the consistency of the binding mode of the ligand at the three pockets of HA in a single MD simulation. The initial pose of M090 in each pocket was manually adjusted to retain the key contacts described by Zhao et al.,^32^ especially regarding the HB formed with T309_1_. At this point, let us note that the residues that shape the binding pocket in this strain are identical to those found in other H1N1 strains (see Homology Modeling, Figure S1, and Table S1 in the Supporting Information (SI)), such as A/Guangzhou/GIRD/07/2009 and A/PR/8/34.

**Figure 2:**
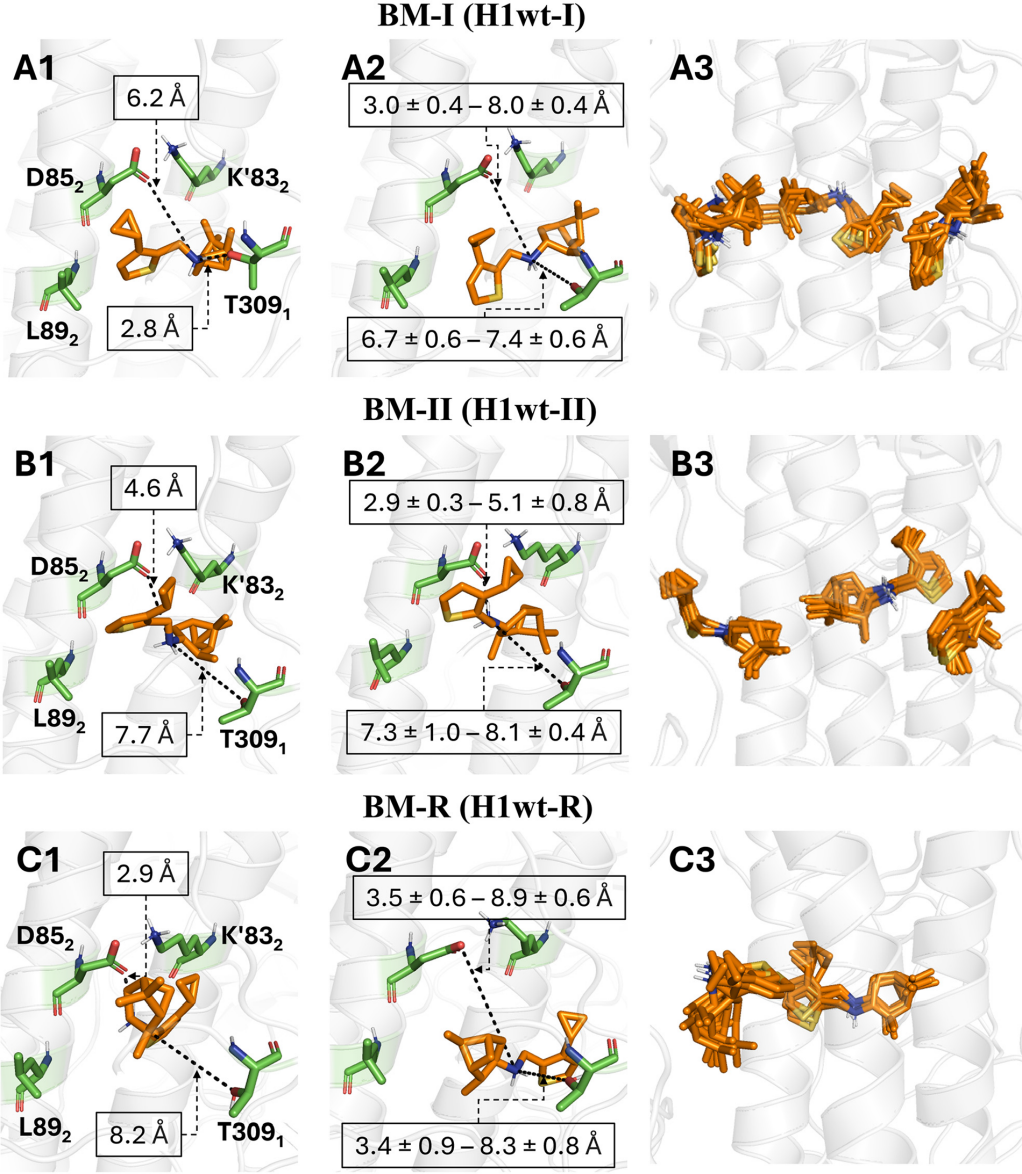
Representation of the three initial binding modes of M090 investigated in this study: (A1) the pose with the restrained HB between the amine nitrogen and T309_1_; (B1) the unrestrained arrangement in the interior of the pocket; (C1) the reversed pose where the thiophene and pinanamine units have been exchanged relative to A1 and B1. Representative snapshots of the poses obtained at the end of the MD simulations with distances to D85_2_ and T309_1_ are shown in the central column (A2, B2, and C2). Finally, superpositions of the ligands taken every 100 ns along the last 500 ns of the MD simulation are displayed in the right column (A3, B3, C3). Note that only two pockets containing overlapped ligands are shown in C3 due to the release of M090 from the third binding pocket. Values denote the distance averaged for the three distinct M090 molecules bound to HA.

Using this model system, two independent MD simulations (H1wt-I and H2wt-I, 1 μs) were run to examine the stability of the BM-I binding mode (see Molecular Dynamics Simulations in the SI). The N–H···O(T309_1_) HB was restrained during equilibration and the first 200 ns of the MD production run to avoid artifactual changes due to structural relaxation during thermalization. The root-mean-square deviation (RMSD) profile determined for the two trajectories supports the structural stability of the binding site (RMSD values ranging from 1.6 to 2.2 Å for the six binding pockets in the two independent MD simulations; Figure S2). However, after release of the restraints, the HB between M090 and T309_1_ was lost in all cases, and the ligand was slightly shifted in the interior of the pocket (RMSD of 2.3–4.9 Å; Figure S2). The new arrangement was stable in five out of six cases (Figure 2A2,A3), i.e., three equivalent binding modes on the HA:M090 (1:3 ratio) complex simulated in two independent MD simulations, with the amine nitrogen being roughly equidistant from residues T309_1_ (distances of 6.7 ± 0.6 to 7.4 ± 0.6 Å) and D85_2_, forming transient salt bridges with this latter residue (distances of 3.0 ± 0.4 to 8.0 ± 0.4 Å). In the remaining pocket, the ligand was released to the bulk solvent. This was facilitated by the displacement of the L2-BS loop, which enabled flipping of the ligand so that the pinanamine ring was facing the mouth of the pocket (shaped by H-BS and L1-BS) at the beginning of the trajectory, and the ligand was finally released at the end of the trajectory (ca. 840 ns).

Because of the previous results, an additional MD simulation (H1wt-II, 2 μs) was run for the HA:M090 (1:3 ratio) complex, where M090 was arranged to mimic the original binding mode (i.e., with the pinane moiety filling the innermost region). However, rather than imposing the HB with T309_1_, the protonated amine was oriented to the carboxylate group of D85_2_, thus defining the binding mode denoted as BM-II (Figure 2B1). The RMSD profiles confirmed the stability of the binding pocket (RMSD of 1.2–1.5 Å) and ligand (RMSD of 1.7–2.7 Å) (Figure S3). M090 adopted a similar pose in the three pockets (Figure 2B2,B3), forming a salt bridge between the amine nitrogen and D85_2_ (distances of 3.1 ± 0.5 to 5.0 ± 0.7 Å for the three cases), which in turn forms an electrostatic interaction with K′83_2_ (distances of 2.9 ± 0.3 to 3.0 ± 0.3 Å) in the H-BS of the neighboring protomer. Let us note that these two residues are preserved in all of the H1N1 strains (see Table S1). The thiophene ring forms van der Waals contacts with the side chains of P307_1_, I308_1_, and L89_2_, and the pinane moiety fills the interior of the pocket through interactions with F′88_2_, A65_2_, and the methylene chain of K′83_2_.

Further support for the BM-II binding mode came from three independent MD simulations (H2wt_II, H3wt-II, and H4wt-II, 1 μs), which were prepared using distinct starting structures (i.e., the homology model and the frames taken at 1 and 2 μs from a simulation of the apo species of HA; see Table 1). M090 was introduced in the center of the three binding sites, with the pinane moiety filling the innermost region and the thiophene ring close to the pocket mouth without imposing restraints with either T309_1_ or D85_2_ (Figure 2B1). The structural integrity of the binding pocket was maintained in all cases (RMSD of 0.8–2.0 Å; Figure S3). Furthermore, the binding mode of M090 was preserved in all of the pockets (RMSD of 1.0–3.6 Å; Figure S3). At this point, let us note that the changes observed in the RMSD profile of the ligand in some trajectories merely reflected reorientations of the pinanamine moiety through rotations around the N–C bond. Finally, the distance from the amine nitrogen to the D85_2_ carboxylate oxygens ranged from 2.9 ± 0.3 to 5.1 ± 0.8 Å for the nine binding sites (three independent MD simulations, each with three molecules of M090 per HA trimer), which supports the electrostatic stabilization of the binding mode exerted by the interaction with D85_2_.

As a final test, we explored the stability of the complex formed with M090 but adopted a reverse binding mode (denoted BM-R) that exchanges the pinane and thiophene moieties in the binding pocket (Figure 2C1). To this end, two additional MD simulations (H1wt-R, 1 μs; H2wt-R, 0.5 μs; Table 1) were run for the ligand in this reverse binding mode without imposing positional restraints. As in the preceding cases, the RMSD profiles supported the structural stability of the binding pocket (RMSD of 1.2–1.6 Å in the two independent MD simulations; Figure S4). After a slight rearrangement, the ligand remained stable in four out of the six cases, i.e., three equivalent binding modes on the HA:M090 (1:3 ratio) complex simulated in two independent MD simulations. However, a precise binding mode was not observed. Thus, in two cases, the protonated amine formed direct interactions with the amide carbonyl oxygen of Q62_2_ (distance of 3.3 ± 1.2 Å; Figure S5), assisted by electrostatic interactions with D′90_2_ (distance of 6.7 ± 1.8 Å). Alternatively, the protonated amine formed a salt bridge with D85_2_ (distance of 3.7 ± 0.8 Å). Finally, in two cases, M090 was released from the binding site, arguing against the feasibility of the reversed binding mode.

The preceding results suggest that M090 adopts a stable binding mode when the pinanamine moiety fills the innermost region of the pocket, as suggested by Zhao et al.,^32^ but with the amine nitrogen forming an electrostatic interaction with D85_2_. To further validate the BM-II binding mode predicted for M090, we planned to perform a series of alchemical transformations taking advantage of the inhibitory potencies reported for a series of M090-related compounds, which affect both the nature of the thiophene ring and the attached cyclopropyl chain (Figure 3A).^32^

**Figure 3:**
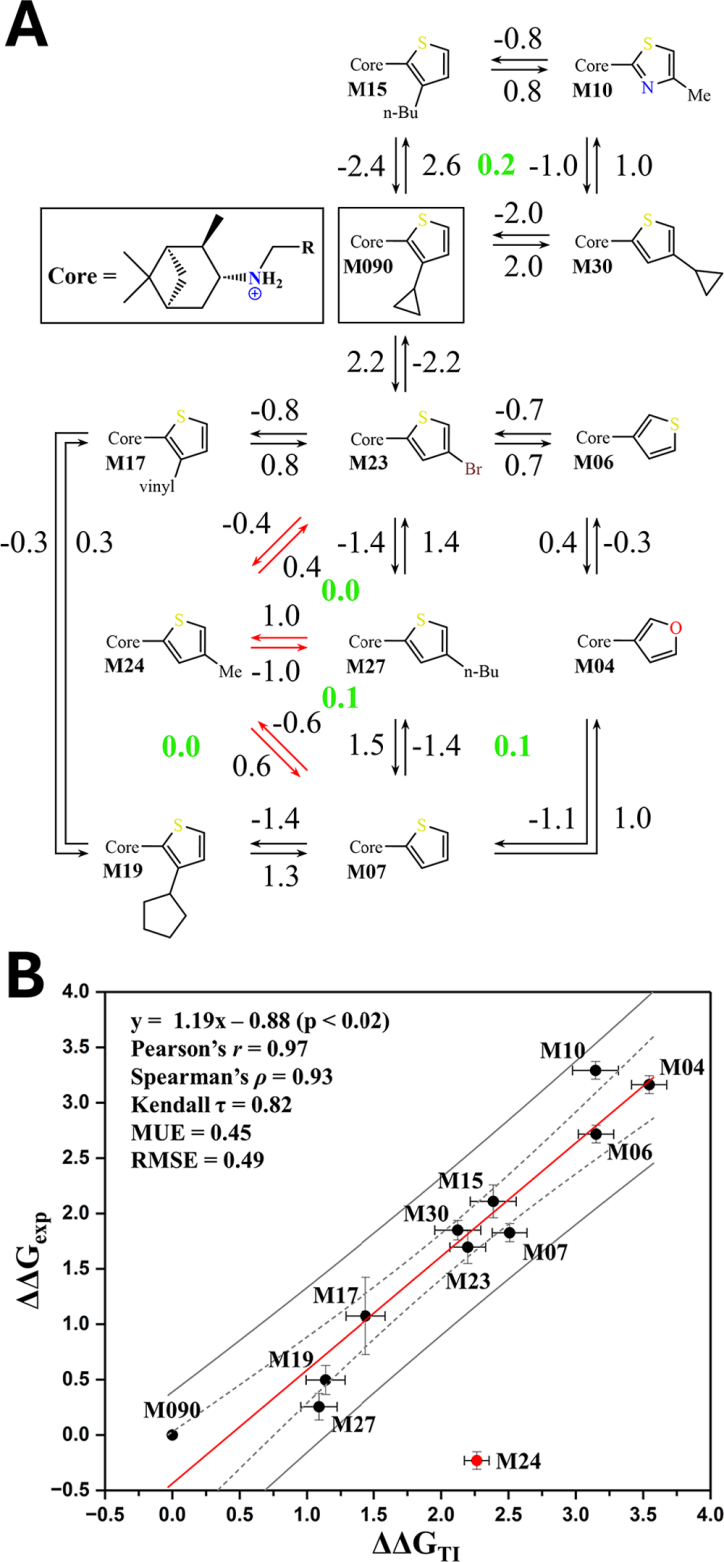
(A) Representation of the free energy changes determined from TI calculations for the alchemical (forward and backward) transformations between M090-related derivatives. The numbering of the derivatives follows the notation used by Zhao et al. in ref (32). Green values denote the sum of the free energy changes for the processes implicated in the closure of the thermodynamic cycle. (B) Comparison between the differences in binding affinity estimated from the experimental EC_50_ data and the relative free energy differences predicted from TI calculations. Bars denote the errors of calculated and experimental relative free energy changes. Dotted and dashed lines denote the 95% confidence and prediction intervals. All values (kcal mol^–1^) are relative to M090. MUE and RMSE stand for mean unsigned error and root-mean-square error, respectively.

Thermodynamic integration (TI) calculations (see Alchemical Calculations in the SI)^34−37^ were carried out to estimate the differences in binding free energy between M090 and its structural analogues. To this end, the free energy changes were determined for the alchemical transformations between ligands bound to HA relative to the same process in an aqueous solution. The changes in the binding free energy were compared with the experimental values, which were approximated from the EC_50_ values reported by Zhao et al.^32^ using the Cheng–Prusoff equation.^38^ With this aim, the inhibitory potency measured from a consistent set of virus (A/Guangzhou/GIRD/07/2009)-infected MDCK cell assays, leading to EC_50_ values ranging from 0.30 μM (M090) to 75 μM (M10; see below), were used.^32^ Let us note that this choice may introduce some discrepancies between the results derived from *in silico* calculations and cell-based assays due to the potential effect of factors unrelated to the binding of the compounds to the putative site in HA.

The free energy changes predicted for the alchemical transformations are shown in Figure 3A. It is worth noting the consistency between the free energy changes determined for the “forward” and “backward” processes for each transformation. Furthermore, the addition of the free energy changes for the combination of processes that define the closure of the thermodynamic cycles is close to zero. Finally, one may also notice the consistency between the free energy differences obtained following distinct pathways that connect M090, which was taken as a reference, to other derivatives relative to M090 (see Table 2). The only exception to this behavior is compound M24, which will be discussed below.

**Table 2:** Free Energy Differences (ΔΔ*G*, in kcal·mol^–1^) in of the Set of Pinanamine-Based Compounds Relative to M090 (Reference Compound^a^)

compound (EC_50_)^b^	path	ΔΔ*G*_TI_^c^
**M04** (60.46 ± 1.75)	M090 → M23 → M06 → M04	3.3
	M090 → M23 → M17 → M19 → M07 → M04	3.5
	M090 → M23 → M27 → M07 → M04	3.2
		**3.3**
**M06** (28.60 ± 0.24)	M090 → M23 → M06	2.9
	M090 → M23 → M17 → M19 → M07 → M04 → M06	3.1
	M090 → M23 → M27 → M04 → M06	**2.9**
		2.9
**M07** (6.42 ± 0.18)	M090 → M23 → M06 → M04 → M07	2.3
	M090 → M23 → M17 → M19 → M07	2.5
	M090 → M23 → M27 → M07	2.2
		**2.3**
**M10** (75.00 ± 0.08)	M090 → M30 → M10	3.0
	M090 → M15 → M10	3.3
		**3.1**
**M15** (10.33 ± 2.17)	M090 → M15	2.5
	M090 → M30 → M10 → M15	2.3
		**2.4**
**M17** (1.82 ± 1.04)	M090 → M23 → M17	1.4
	M090 → M23 → M06 → M04 → M07 → M19 → M17	1.2
	M090 → M23 → M27 → M07 → M19 → M17	1.1
		**1.2**
**M19** (0.69 ± 0.12)	M090 → M23 → M17 → M19	1.1
	M090 → M23 → M06 → M04 → M07 → M19	0.9
	M090 → M23 → M27 → M07 → M19	0.8
		**0.9**
**M23** (5.16 ± 1.08)	M090 → M23	2.2
**M24** (0.20)	M090 → M23 → M27 → M24	1.8
	M090 → M23 → M06 → M04 → M07 → M24	2.9
	M090 → M23 → M06 → M04 → M07 → M27 → M24	3.3
	M090 → M23 → M17 → M19 → M07 → M24	1.8
	M090 → M23 → M17 → M19 → M07 → M27 → M24	1.9
		**1.8**
**M27** (0.46 ± 0.07)	M090 → M23 → M06 → M04 → M07 → M27	0.9
	M090 → M23 → M17 → M19 → M07 → M27	1.0
	M090 → M23 → M27	0.8
		**0.9**
**M30** (6.68 ± 0.42)	M090 → M30	2.0
	M090 → M15 → M10 → M30	2.3
		**2.1**

a EC_50_ = 0.30 ± 0.04 μM.

b Experimental EC_50_ values (μM) taken from ref (32).

c Values averaged for distinct pathways are highlighted in bold.

Figure 3B shows the comparison between the differences in the binding free energy estimated from experimental data and the values predicted from TI calculations. The results reveal that there is a close correspondence between predicted and experimental values, which is remarkable keeping in mind the potential uncertainties arising from the virus-infected MDCK cell assays. Likewise, the error between predicted and experimentally derived values amounts to 0.5 kcal/mol.

The TI results point out the relevance of the 3-cyclopropylthiophene unit to the inhibitory activity. This may be attributed to a hydrophobic shielding effect,^39−42^ which prevents the access of water molecules to the interior of the binding pocket, where they could compete with the salt bridge formed between the amine nitrogen and the carboxylate group of D85_2_. In this context, replacement of the cyclopropyl unit by a linear *n*-butyl chain (compound M15; EC_50_ of 10.33 μM) leads to a 33-fold reduction in the inhibitory potency, which reflects the enhanced steric clashes with G67_2_ at the L2-BS loop (Figure 4A), together with a larger conformational penalty of the flexible butyl chain compared to the more rigid cyclopropyl unit, which only has one rotatable bond. In contrast, the inhibitory potency of M090 remains almost unaltered upon translation of the *n*-butyl chain from position 3 (M15) to 4 (M27; EC_50_ of 0.46 μM), as this reorients the aliphatic chain toward the mouth of the pocket, adopting a more extended conformation. This permits the avoidance of the steric clashes with L2-BS and favors the formation of van der Waals contacts with P307_1_ and I308_1_ in L1-BS and the methylene chain of K82_2_ in H-BS (Figure 4B). Similarly, elimination of the alkyl chain (M07; EC_50_ of 6.42 μM) reduces the hydrophobic shielding, which would justify the 21-fold decrease in potency.

**Figure 4:**
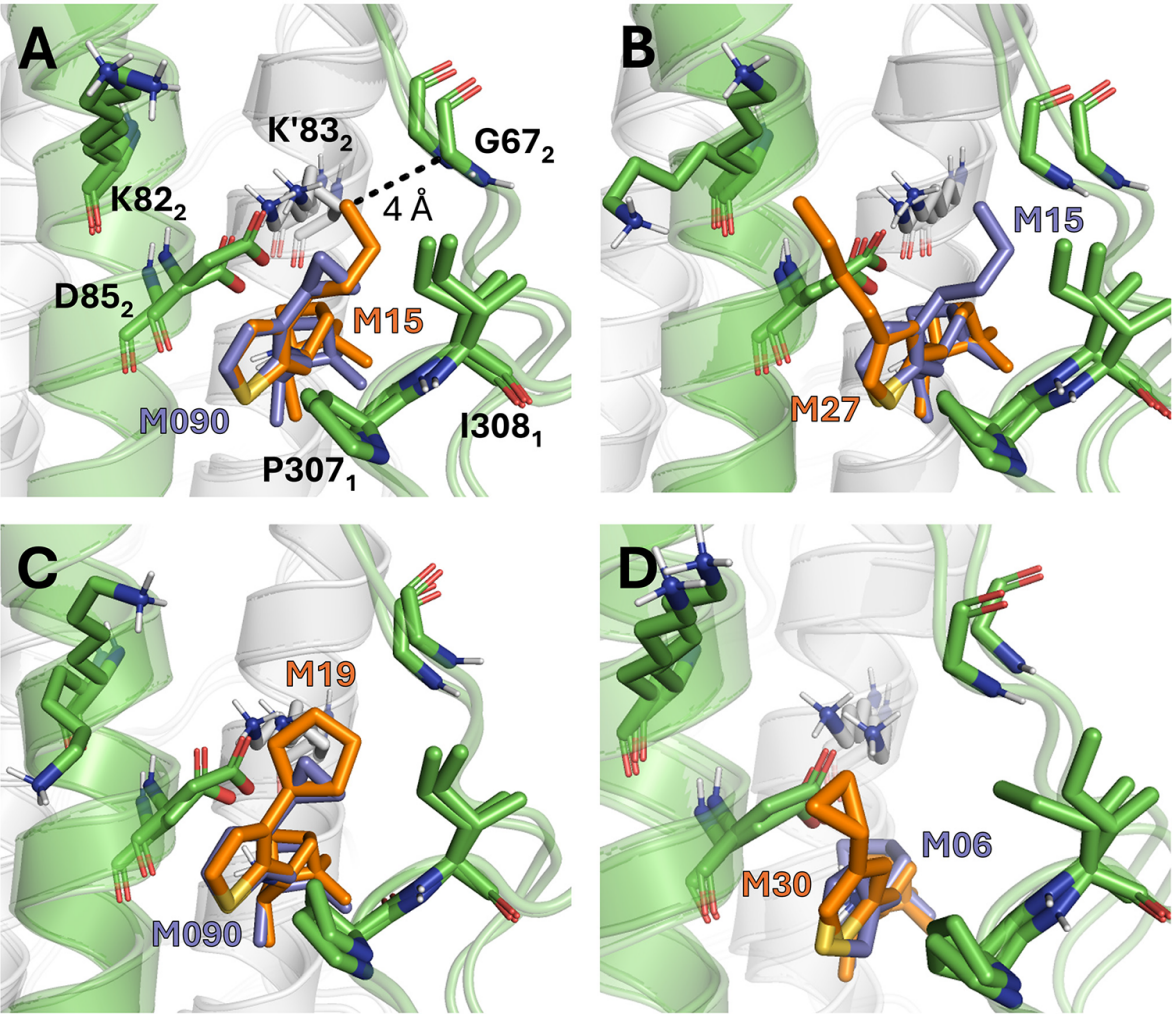
Representative snapshots of the binding pose obtained for compounds (A) M15, (B) M27, (C) M19, and (D) M30 (shown as sticks) at the end of the alchemical transformations. (A) The *n*-butyl chain attached to position 3 of the thiophene unit adopts a folded conformation due to the steric clash with loop L2-BS. (B) Superposition of the complexes with *n*-butyl derivatives M15 (bound to position 3) and M27 (attached to position 4). The steric clash observed for M15 with L2-BS is alleviated in M27, where the *n*-butyl chain adopts an extended conformation and forms van der Waals contacts with P307_1_, I308_1_, and the side chain of K82_2_. (C, D) The cyclopentyl unit in M19 occludes the access of water molecules, thus creating hydrophobic shielding of the electrostatic interaction between D85_2_ and K83_2_ in the neighboring protomer, mimicking the protecting effect of the cyclopropyl unit in M090. This effect is lost in compounds M04, M06, and M07, which have unsubstituted furan and thiophene rings.

The results also point out the feasibility of accommodating the cyclopentyl unit (M19; EC_50_ of 0.69 μM) instead of the cyclopropyl one, which agrees with the ∼2-fold reduction in inhibitory potency (Figure 4C). Finally, the relevance of the cyclopropyl chain is also reflected in the loss of potency generated by changing the linkage between the pinanamine and thiophene moieties from position 2 in M090 to position 3 in M30 (EC_50_ of 6.68 μM) and even upon deletion of the cyclopropyl unit in M06 and M07 (EC_50_ of 28.60 and 6.42 μM, respectively), thus suppressing the hydrophobic shielding due to the larger accessibility of waters to the interior of the pocket (Figure 4D). This also justifies the drastic reduction in potency observed upon replacement of the thiophene ring by more polar moieties, such as furan and thiazole, leading to 200-fold (M04; EC_50_ of 60.46 μM) and 250-fold (M10; EC_50_ of 75 μM) reductions in inhibitory potency, respectively.

The only exception to this behavior is compound M24 (red dot in Figure 3B), which is predicted to be much less active compared to the experimental EC_50_ (0.20 μM). As noted in Figure 3A, consistent trends were obtained in alchemical transformations leading from M23, M27, and M07 to M24. For instance, shortening of the *n*-butyl chain (M27) to methyl (M24) should destabilize the hydrophobic shielding effect, whereas insertion of methyl in M07 to generate M24 should increase the shielding effect. We speculate that the activity of M24, which is slightly more potent than M090, may reflect the ability to interact with another viral target, particularly the M2 proton channel.^43^ In this regard, one may notice the close hydrophobic/philic-guided overlap determined with Pharmscreen^44^ between M24 and *N*-[(5-bromothiophen-2-yl)methyl]adamantan-1-amine,^45^ a compound that showed ∼77% inhibition of the wild-type M2 channel and its S31N mutant (EC_50_ of 1.8 mM against the A/WSN/33 (S31N) strain).^46^ Indeed, superposition of the overlapped molecules in the X-ray crystallographic structure of the complex formed by this compound with the S31N mutant (PDB ID 2MUV) reflects a fine accommodation in the pore of the channel (Figure 5). However, severe steric clashes can be anticipated upon attachment of the cyclopropyl unit at position 3 of the thiophene ring in M090 (Figure 5), which agrees with the lack of inhibition against the M2(S31N) channel reported by Zhao et al.^32^

**Figure 5:**
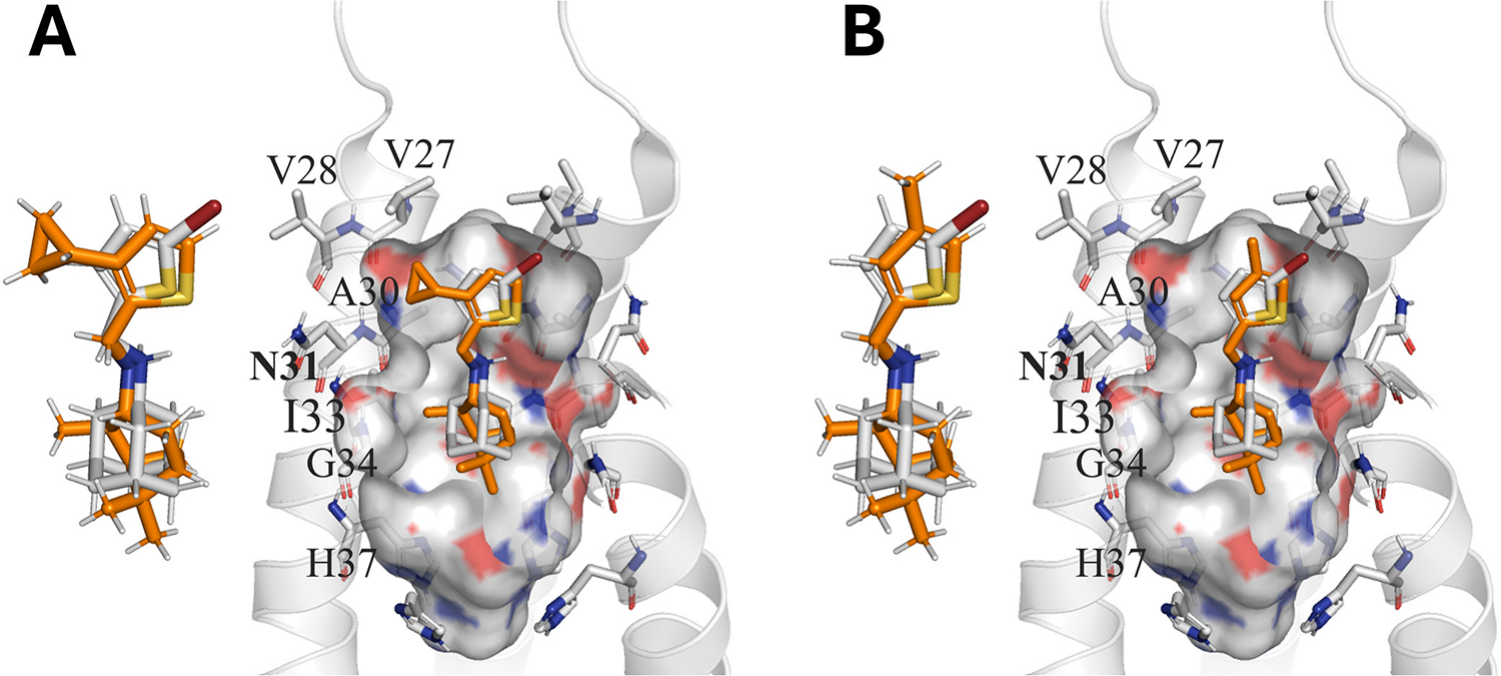
Overlaid structures of (A) M090 and (B) M24 (orange sticks) with *N*-[(5-bromothiophen-2-yl)methyl]adamantan-1-amine (gray sticks) and superposition of the overlaid compounds onto the X-ray crystallographic structure of the S31N mutated variant of the M2 proton channel (PDB ID 2MUV).^46^

Finally, to further assess the feasibility of the binding site proposed for M090, we examined the effect of the drug-resistant mutation E74_2_ → D disclosed from resistance-induction assays using the A/Guangzhou/GIRD/07/2009 (H1N1) virus in MDCK cells.^32^ It is noteworthy that this is the sole mutation found in the mature M090-resistant virus strain. E74_2_ is placed at the top of the α-helix (H-BS) of the HA_2_ domain, where it forms a salt bridge with R76_2_ (Figure 1). Although E74_2_ is distant from the binding pocket, as noted in the distances from the Cα atom of E74_2_ to the Cα atoms of D85_2_ and T3091 (∼17 and 24 Å, respectively; see Table S2), the salt bridge is located at the hinge between H-BS and L2-BS, and hence, its stability may affect the structural flexibility of the binding pocket.

To check the impact of the E74_2_ → D mutation, MD simulations were run for the wild type and mutated variants of apo HA (Awt and Amut, 2 μs; Table 1). The RMSD profiles support the structural stability of the binding pocket (RMSD values ranging from 1.1 to 2.4 Å; Figure S8). As expected, the salt bridge between E74_2_ and R76_2_ is almost fully maintained in the wild type species (average population of 96%), reflecting primarily a bidentate interaction between the carboxylate oxygens and guanidinium NH groups. However, its population is reduced to ∼50% in the mutated species. Similar trends were observed in the comparison of MD simulations run for the wild type holo species (H1wt-II, H2wt-II, H3wt-II, and H4wt-II) and two MD simulations run by placing M090 in the three pockets of the mutated HA preserving the binding pose BM-II (H1mut-II and H2mut-II, 1 μs; Table 1; see Figure S9 for the RMSD profiles of the holo complexes run for the mutated HA). The salt bridge between E74_2_ and R76_2_ is observed in 87–95% of the four trajectories run for the wild type M090-bound HA. However, this interaction is reduced to 46% and 54% in the two independent MD simulations of the mutated holo species. It is noteworthy that the electrostatic destabilization caused by the E74_2_ → D mutation also affects secondary interactions, such as the contact between R76_2_ and E69_2_, which is located in L2-BS. Thus, this contact is found, on average, in 66% of the snapshots analyzed for the apo and holo wild type simulations, but it is reduced to 41% in the mutated strain.

Principal component (PC) analysis was used to examine the impact of the E74_2_ → D mutation on the motions of the backbone atoms of H-BS, L1-BS, and L2-BS in the apo wild type and mutated HA. The first two PCs explain ∼50% of the total variance (the contributions of higher-order components are lower than 7%). The first PC accounts for 30% of the structural variance and describes a rotational motion of H-BS, L1-BS, and L2-BS, following the *C*_3_ symmetry axis of HA (Figure 6A). In contrast, the second PC (19%) describes a conformational remodeling of L2-BS. In the case of the holo species, the two main essential motions (37% and 18% of the structural variance) exhibit a large resemblance to corresponding motions found for the apo species (Figure 6A).

**Figure 6:**
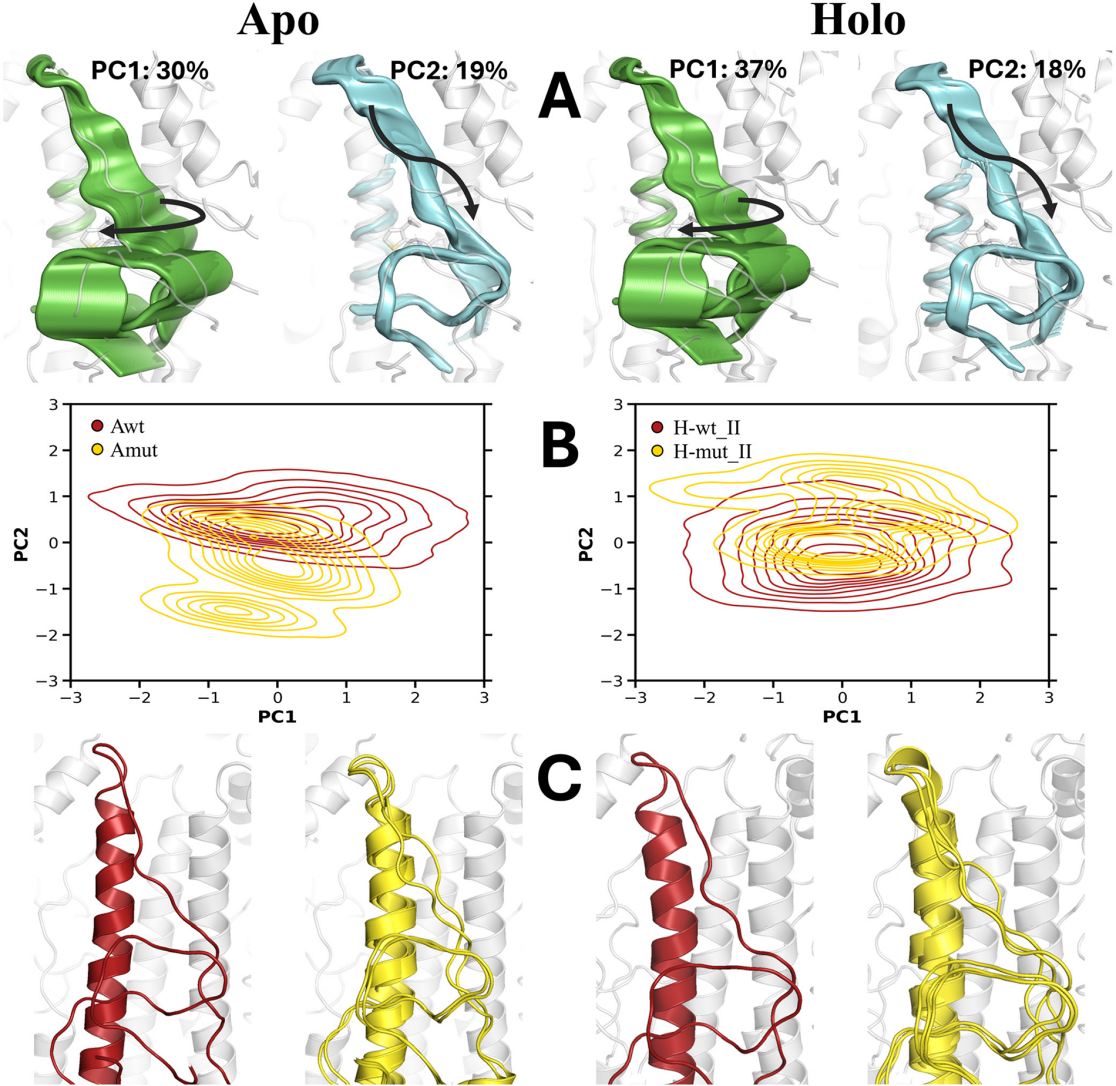
(A) Representation of the two main essential motions of the backbone atoms that shape the binding pocket for the (left) apo and (right) holo species of wild type and E74_2_ → D mutated HA. (B) Projections of the snapshots sampled for the apo wild type HA (red) and its mutated variant (yellow). (C) Centroids of the clusters observed for the apo wild type and mutated HA (backbone of the binding pocket in red and yellow, respectively).

The projections of the snapshots collected from the simulations of wild type HA and the mutated variant reveal that the main difference involves the second PC (Figure 6B). Indeed, whereas a single cluster is observed for the wild type HA in both the apo and holo forms, two (apo) and three (holo) clusters are found in the projection of the snapshots sampled for the mutated HA (Figure 6C). This reflects that the E74_2_ → D mutation induces a larger conformational flexibility in the L2-BS loop, which should alter the packing against H-BS and L1-BS and weaken the proper accommodation of M090 in the binding pocket. Furthermore, we cannot rule out larger spatial rearrangements on longer time scales, especially upon acidification.

Overall, this study provides support for the involvement of the putative binding site proposed by Zhao et al.^32^ for the binding of M090. MD simulations reveal a stable binding pose characterized by the insertion of the pinanamine moiety in the innermost region of the pocket, whereas the 3-cylopropylthiophene ring occupies the mouth of the pocket, occluding the accessibility of water molecules. Instead of the HB formed between the amine nitrogen and T309_1_, our results support the electrostatic stabilization of the protonated nitrogen with D85_2_, a residue conserved in all the H1N1 strains considered in the experimental assays. Furthermore, this binding mode provides a structural basis to justify the effect of the chemical changes introduced in both the cyclopropyl and thiophene units, since the alchemical transformations discussed here generally exhibit a close correspondence with the differences in inhibitory potency determined in antiviral assays. Finally, as hypothesized by Zhao et al.,^32^ the E74_2_ → D mutation has a negative impact on the stability of the salt bridge formed with R76_2_, which in turn also affects other secondary interactions, leading ultimately to an increase in the structural flexibility of the L2-BS loop. Taken together, these results support the occurrence of a binding pocket alternative to sites A and B in HA (Figure 1), which may be valuable to explore the fusion inhibition by other compounds with an unknown mechanism of action.

## Supporting Information Available

Details of MD simulations, thermodynamic integration calculations, and supplementary figures (PDF)

## Abbreviations

FP: fusion peptide
HA: hemagglutinin
HB: hydrogen bond
MD: molecular dynamics
PC: principal component
RMSD: root-mean-square deviation
SA: syalic acid
TI: thermodynamic integration
