# Supplementary material for: Mining Druggable Sites in Influenza A Hemagglutinin: Binding of the Pinanamine-Based Inhibitor M090
Source: ACS Med Chem Lett. 2024 Nov 28;16(1):126–35. doi: 10.1021/acsmedchemlett.4c00502 (PMC11726360; doi:10.1021/acsmedchemlett.4c00502)
Supplement: Supplementary file 1 — ml4c00502_si_001.pdf [file ml4c00502_si_001.pdf]

# Supporting Information to

# Mining druggable sites in influenza A hemagglutinin: Binding of the pinanamine-based inhibitor M090

Aitor Valdivia,[†,‡] Maria Rocha,[†,¶] F. Javier Luque[†,‡,§,*]

[†] Departament de Nutrició, Ciències de l'Alimentació i Gastronomia, Facultat de Farmàcia i Ciències de l'Alimentació - Campus Torribera, Universitat de Barcelona, Prat de la Riba 171, 08921 Santa Coloma de Gramenet, Spain

[‡] Institut de Biomedicina (IBUB), Universitat de Barcelona

[¶] Department of Life Sciences, University of Coímbra, Calçada Martim de Freitas, 3000-456 Coímbra, Portugal

[§] Institut de Química Teòrica i Computacional (IQTCUB), Universitat de Barcelona

[*] Email: fjluque@ub.edu

**Index**

| | |
|---|---|
| Homology modeling | S2 |
| Figure S1 | S2 |
| Table S1 | S3 |
| Molecular Dynamics simulations | S4 |
| Figure S2 | S6 |
| Figure S3 | S7 |
| Figure S4 | S8 |
| Figure S5 | S9 |
| Alchemical transformations | S10 |
| Figure S6 | S13 |
| Figure S7 | S14 |
| Principal component analysis | S15 |
| Table S2 | S15 |
| Figure S8 | S16 |
| Figure S9 | S17 |
| References | S18 |

*Homology modelling*

The 3D structure of HA (H1N1 A/Virginia/ATCC3/2009) was modelled using SWISS-MODEL[S1] and the crystallographic structure of the A/PR/8/34 H1 HA as structural template (PDB ID 6WCR,[S2] resolution 2.68 Å). This was chosen to take advantage of previous studies focused on the interaction of aniline-based antiviral compounds with HA.[S3] Furthermore, the residues that shape the pocket proposed for the binding of M090 are preserved in distinct H1N1 HA subtypes (see Table S1). Thus, comparison of the sequence content of the α-helix formed by $L80_2$–$A96_2$ (H-BS) and the loops $P301_1$–$K318_1$ and $M59_2$–$L73_2$ (L1-BS and L2-BS, respectively) for A/Virginia/ATCC3/2009, A/California/04/2009, A/Texas/04/2009, A/Guangzhou/GIRD/07/2009, A/California/07/2009, A/WSN/1933 and A/PR/8/1934 shows very few changes, such as the replacement of $K312_1$ by E in A/WSN/1933 and A/PR/8/1934, $T61_2$ and $H72_2$ by I and K in A/PR/8/193, and $D86_2$ by G in A/Texas/04/2009. Most of these changes occur in solvent-exposed areas of the binding pocket (Figure S1). In contrast, larger changes are observed in H3N2, H7N3 and H9N2 strains, which may contribute to the weakening in the inhibitory potency.

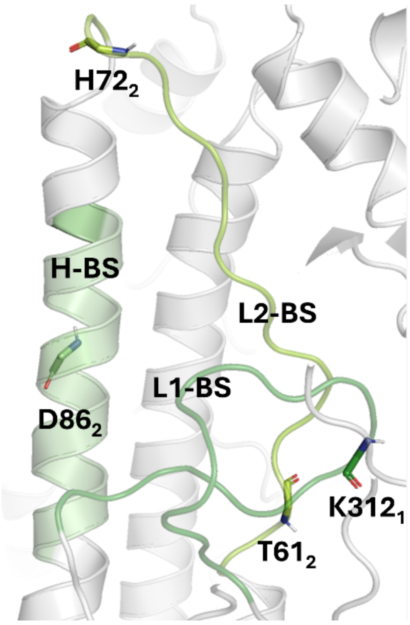


**Figure S1**. Representation of the structural elements that shape the putative binding pocket of M090 in HA: the α-helix formed by $L80_2$–$A96_2$ (H-BS) and the loops $P301_1$–$K318_1$ and $M59_2$–$L73_2$ (L1-BS and L2-BS, respectively). The location of the residues affected by the few non-conservative mutations between the viral H1N1 strains are highlighted with the backbone represented as sticks.

**Table S1.** Sequence alignment of the structural elements that shape the proposed binding pocket of M090 in hemagglutinin: the α-helix formed by $L80_2$–$A96_2$ (H-BS) and the loops $P301_1$–$K318_1$ and $M59_2$–$L73_2$ (L1-BS and L2-BS, respectively). Conservative changes in the nature of the residues are highlighted in green, whereas non-conservative changes are shown in red.

| HA strain | $EC_{50}$ (μM) | Sequence (**L1-BS**) | Sequence (**L2-BS**) | Sequence (H-**BS**) |
|---|---|---|---|---|
| A/Virginia/ATCC3/2009 (H1N1) | --- [a] | **PFQNIHPITIGKCPKYVK** | **MNTQFTAVGKEFNHL** | **LNKKVDDGFLDIWTYNA** |
| A/California/04/2009 (H1N1) | --- [b] | **PFQNIHPITIGKCPKYVK** | **MNTQFTAVGKEFNHL** | **LNKKVDDGFLDIWTYNA** |
| A/Texas/04/2009 (H1N1) | 0.10 [c] | **PFQNIHPITIGKCPKYVK** | **MNTQFTAVGKEFNHL** | **LNKKVDGGFLDIWTYNA** |
| A/Guangzhou/GIRD/07/2009 (H1N1) | 0.30 [d] | **PFQNIHPITIGKCPKYVK** | **MNTQFTAVGKEFNHL** | **LNKKVDDGFLDIWTYNA** |
| A/California/07/2009 (H1N1) | 0.34 [c] | **PFQNIHPITIGKCPKYVK** | **MNTQFTAVGKEFNHL** | **LNKKVDDGFLDIWTYNA** |
| A/WSN/1933 (H1N1) | 1.48 [e] | **PFQNIHPVTIGECPKYVR** | **MNTQFTAVGKEFNNL** | **LNKKVDDGFLDIWTYNA** |
| A/PR/8/1934 (H1N1) | 2.23 [e] | **PYQNIHPVTIGECPKYVR** | **MNIQFTAVGKEFNKL** | **LNKKVDDGFLDIWTYNA** |
| A/Aichi/2/1968 (H3N2) | 4.55 [e] | **PFQNVNKITYGACPKYVK** | **TNEKFHQIEKEFSEV** | **LEKYVEDTKIDLWSYNA** |
| A/duck/Guangdong/1/1996 (H7N3) | 4.58 [e] | **PFQNINPRTVGRCPRYVK** | **TNQQFELIDNEFSEI** | **VINWTRDSMTEVWSYNA** |
| A/Hong Kong/1968 (H3N2) | 5.38 [e] | **PFQNVNKITYGACPKYVK** | **TNEKFHQIEKEFSEV** | **LEKYVEDTKIDLWSYNA** |
| A/duck/HK/Y280/1997 (H9N2) | 6.79 [e] | **PFHNVSKYAFGNCPKYVG** | **MNKQYEIIDHEFSEV** | **INNKIDDQIQDIWAYNA** |

[a] Structural model used in this work. [b] Structural used in ref. S3. [c] Activity measured in plaque reduction assay. [d] Activity determined in virus-Infected MDCK cells. [e] Activity measured in cytophatic effect assay.

*Molecular Dynamics simulations*

The amberff14sb force field[S4] was used for the protein, and M090 and its derivatives were parameterised using the gaff2 force field.[S5] Partial charges for the ligands were derived using the RESP protocol[S6] at the B3LYP/6-31G(d) level of theory, as calculated using Gaussian16.[S7] The systems were solvated with a truncated octahedral box of TIP3P[S8] water molecules, allowing a distance of 12 Å from the protein to the box edge. Counterions ($K^+$, $Cl^-$)[S9] were added to maintain the neutrality of the simulated system and to keep the ion concentration at 0.15 M using the SPLIT method.[S10] The simulated systems contain ~246,000 atoms.

Each system was minimised using 7500 steps of steepest descent, followed by 7500 steps of conjugate gradient algorithm. Equilibration was performed in 3 steps. The systems were slowly heated in the NVT ensemble from 5 K to 300 K in a temperature ramp for 250 ps and held at 300K for 50 ps imposing positional restraints (5 $kcal \cdot mol^{-1}$) on the ligands. The density of the system was then equilibrated for 4.7 ns in the NPT ensemble (pressure: 1 bar, T: 300 K). Finally, after density equilibration, the system was simulated using the NVT ensemble for 1 ns and prepared for the MD production.

During the production runs, temperature was controlled using Langevin dynamics with a collision frequency of 2 $ps^{-1}$, and pressure was maintained using the Berendsen barostat during the density equilibration step. All bonds involving hydrogen atoms were constrained by the SHAKE algorithm[S11] to allow a timestep of 2 fs. Since the X-ray structural information is limited to the ectodomain, a series of distance NMR restraints were applied to avoid an artefactual increase of flexibility at the C-termini of the HA trimer. In particular, restraints (5 $kcal \cdot mol^{-1}$) were applied to the $C_\alpha$ of two residues ($N128_2$ and $R170_2$) of each HA monomer when a displacement greater than 2Å from the crystallographic distance occurred (the restraint is applied progressively until the restraint is fixed at 1.5 Å beyond 2Å from the crystallographic distance, i.e., at 3.5 Å from the crystallographic distance). All simulations were performed using the AMBER20 package.[S12]

The structural flexibility of the binding pocket and the stability of M090 was assessed through the analysis of a variety of MD simulations for the apo and holo systems (Table 1). The initial model was simulated in the apo state for 2 μs of unbiased MD.

For the ligand-bound complexes, three binding modes were considered (Figure S2). The first one corresponds to the binding pose reported by Zhao *et al.,*[S13] which will be denoted

BM-I and is characterized by the formation of the hydrogen bond between the amine nitrogen of M090 and $T309_1$. The second binding mode (BM-II) is characterized by the formation of a salt bridge between the charged nitrogen of M090 and $D85_2$. Finally, the third binding pose (BM-R) is characterized by the exchange of the pinanamine and thiophene units in the binding pocket. The holo systems were simulated using at least two independent MD simulations, each simulated for 1 μs (the only exception is one of the simulations performed for the BM-R binding mode, which was run for 0.5 μs due to the release of one of the ligands from the binding pocket). In one case the starting structure was the equilibrated HA model obtained from the SWISS MODEL web server. Alternatively, the starting structure was the frame obtained after 1 or 2 μs of the MD simulation run for the apo system.

Finally, the apo species of the $E74 \rightarrow D74_2$ mutant was simulated for a total of 2 μs, and the ligand-bound complex formed with the $E74 \rightarrow D74_2$ mutant was examined considering two MD simulations extended up to 1 μs each.

**Figure S2**. RMSD profiles determined for (left) the backbone of the residues that define the three binding pockets and (right) the bound ligand in the two MD simulations (H1wt-I and H2wt-I) run for the complex between M090 (binding mode BM-I) and HA.

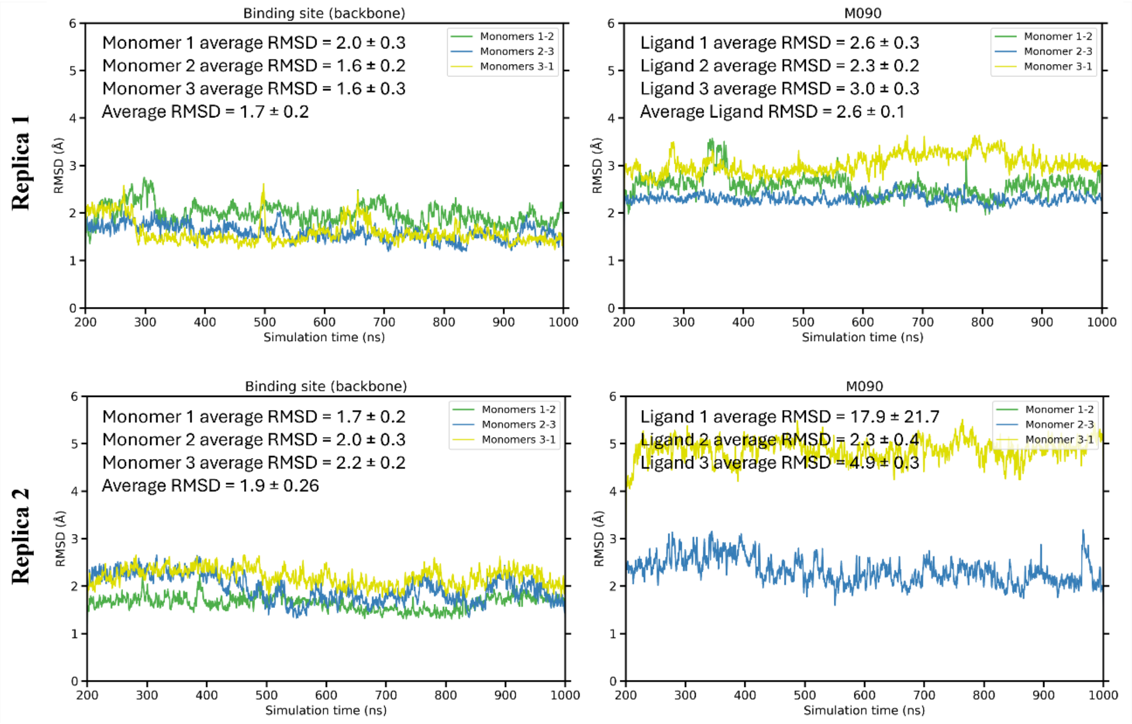

**Figure S3**. RMSD profiles determined for (left) the backbone of the residues that define the three binding pockets and (right) the bound ligand in the four MD simulations (H1wt-II, H2wt-II, H3wt-II and H4wt-II) run for the complex between M090 (binding mode BM-II) and HA.

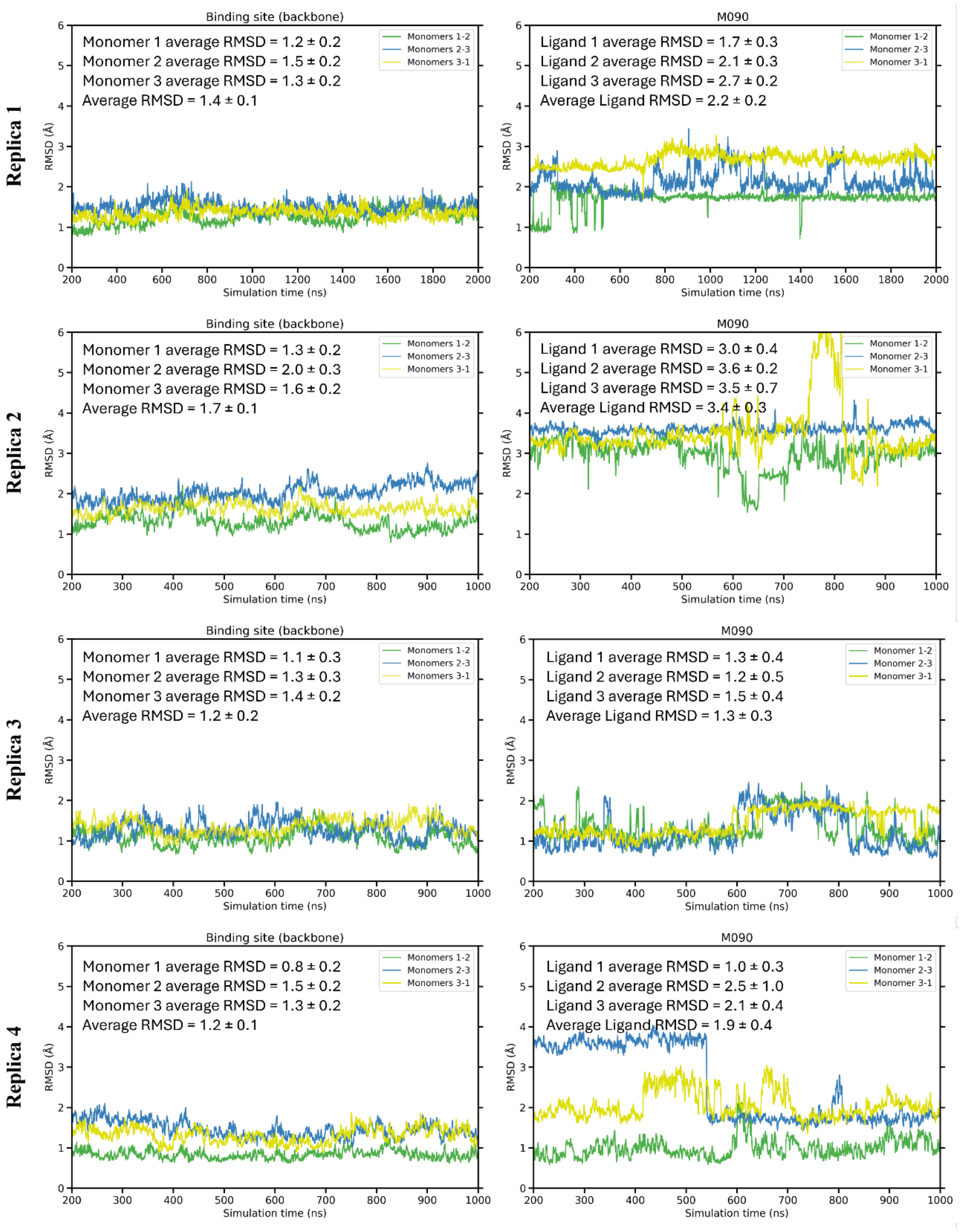

**Figure S4**. RMSD profiles determined for (left) the backbone of the residues that define the three binding pockets and (right) the bound ligand in the two MD simulations (H1wt-R and H2wt-R) run for the complex between M090 (binding mode BM-R) and HA.

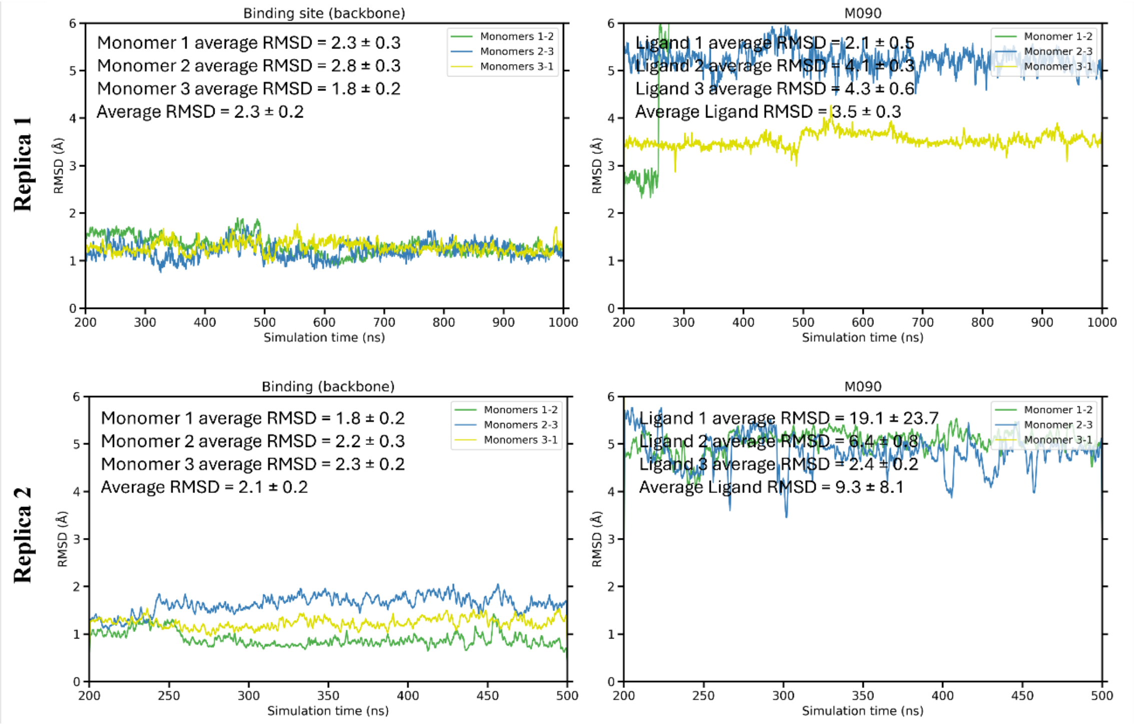

**Figure S5**. Representation of the alternative binding mode found for M090 in the simulations run for the reversed binding mode (H1wt-R and H2wt-R), where the protonated amine formed direct interactions with the amide carbonyl oxygen of $Q62_2$, assisted by electrostatic interactions with $D'90_2$.

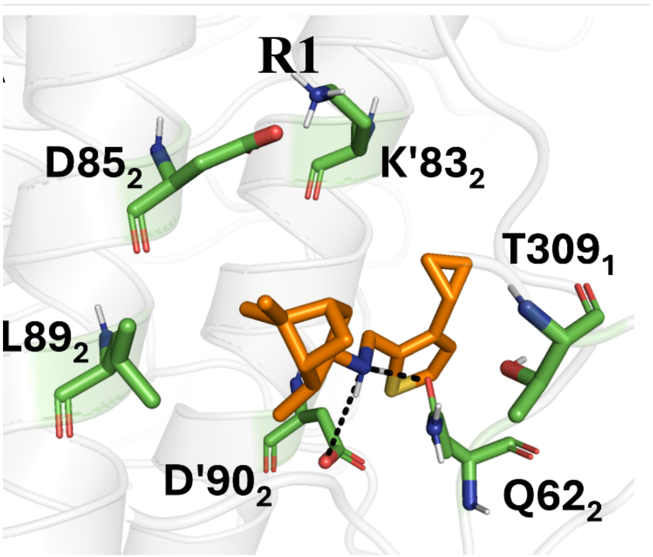

*Alchemical calculations*

The relative binding free energy (RBFE)[S14] between derivatives of M090 was determined by alchemical transformations, where a ligand ($L_1$) is converted to a structurally related analogue ($L_2$) both in the protein-bound complex and in the unbound state in aqueous solution (Eq. 1).

$$\Delta\Delta G_{bind} = \Delta\Delta G_{complex}(L_1 \rightarrow L_2) - \Delta\Delta G_{water}(L_1 \rightarrow L_2) \quad (1)$$

The transformation from $L_1$ to $L_2$ was divided into a series of windows, where the states corresponding to λ = 0 and λ = 1 represent the holo species with $L_1$ and $L_2$, respectively, and the intermediate λ values denote a linear interpolation between the parameters of the initial and final systems. Alchemical transformations were performed using 29 equally spaced λ windows, including states λ = 0 and λ = 1 (i.e., λ = 0.000000, 0.035714, 0.071429, 0.107143, 0.142857, 0.178571, 0.214286, 0.250000, 0.285714, 0.321429, 0.357143, 0.392857, 0.428571, 0.464286, 0.500000, 0.535714, 0.571429, 0.607143, 0.642857, 0.678571, 0.714286, 0.750000, 0.785714, 0.821429, 0.857143, 0.892857, 0.928571, 0.964286, 1.000000).

To smoothly transform $L_1$ into $L_2$, we followed a concerted alchemical transformation protocol using a dual-topology scheme. During the transformation, two copies of the common core (CC) of the molecules are present with identical sets of atomic coordinates to facilitate phase space overlap between states during the alchemical transformation. The non-common regions for each pair of molecules (i.e., the atoms with different sets of coordinates) were evaluated with soft-core potentials.[S15] All the λ simulations were run for 12ns - 18ns, depending on the needs to reach the convergence criteria. Calculations were performed using the GPU-accelerated TI implementation of AMBER20.

The Thermodynamic Integration (TI) estimator as implemented in *alchemlyb* was used to estimate the free energy change for each λ simulation. In addition, each transformation was simulated in both directions to assess hysteresis. For each alchemical transformation, we determined the average value of $< \frac{dH}{d\lambda} >_i$ and the standard error (and variance) at each lambda. The free energy difference between initial and final states was estimated through integration of the set of $< \frac{dH}{d\lambda} >_i$ values using the trapezoidal rule. Similarly, the error was estimated from the propagation of the variances determined at each lambda between the initial and final states. Since each alchemical transformation was performed in forward and backward directions, the free energy change was estimated as the average of the two

values, and the error was determined as the square root of the sum of the corresponding variances.

In the study, all the free energy differences are given relative to M090, which was used as reference. Accordingly, when comparison of a given compound relative to M090 involves several alchemical transformations (i.e, M090 → M23 → M06 → M04; see Figure 3), the global free energy difference was determined from the addition of the free energy changes for each individual transformation, and the global error from the cumulative propagation of the variances.

In few instances several pathways may be used to link a given compound to M090. For instance, the transformation M090→M04 may be completed using 3 possible paths following the perturbation map in Figure 3:

1. M090 → M23 → M06 → M04
2. M090 → M23 → M17 → M19 → M07 → M04
3. M090 → M23 → M27 → M07 → M04

In this case, the final free energy difference was taken as the average value of the 3 values corresponding to each path, and the final error was estimated from the square root of the sum of the variances for the distinct paths.

The initial coordinates of the system were extracted from the H1wt- II simulation at 1 μs (Table 1), keeping the positions of the water molecules at 8 Å of each M090 molecule to improve the reproducibility of the binding mode during the equilibration of the system after changing the topology. All the systems were then carefully equilibrated with the dual topology using the TI code for each transformation with its respective λ value. Each system was first heated in the NVT ensemble from 5 K to 300 K (150 ps), equilibrated at 1 bar in the NPT ensemble (300 ps), and finally switched back to the NVT (100 ps) ensemble to avoid artifacts at the beginning of the TI simulations.

During the equilibration protocol, the heavy atoms of the protein and ligand were restrained with positional restraints (10 $kcal \cdot mol^{-1}$) to maintain the binding mode obtained during the MD. Each λ state was then simulated in the NVT ensemble at 300 K using the Langevin thermostat and the SHAKE algorithm for bonds involving hydrogen. The analysis and integration of the final free energy values was performed using the last 6ns of the total sampling for each λ state.

The experimental binding free energies, $\Delta\Delta G_{exp}$, were approximated from the experimental $EC_{50}$ reported by Zhao *et al.*[S13] using influenza virus-infected MDCK cell assays (Eq. 2). It should be noted that this choice may introduce some discrepancies

between the results from in silico calculations and cell-based assays due to the potential effect of other factors not directly related to the binding of the compounds to the putative binding site, such as the effective concentration of the ligand and non-specific binding events, in the experimental assays.

$$\Delta\Delta G_{\text{exp}} = -RTln\frac{EC_{50}(L_2)}{EC_{50}(L_1)} \quad (2)$$

where $\Delta\Delta G_{\text{exp}}$ is in kcal·mol$^{-1}$, R ≈ 1.987·10$^{-3}$ kcal·mol$^{-1}$·K$^{-1}$ and T is the temperature in K (300K).

Figures S6 and S7 show representative plots used in the analysis of the free energy changes determined from TI calculations for the alchemical transformations between M04 and M06 in aqueous solution and in the protein-ligand complex.

**M04 → M06**

**8.244 ± 0.019**

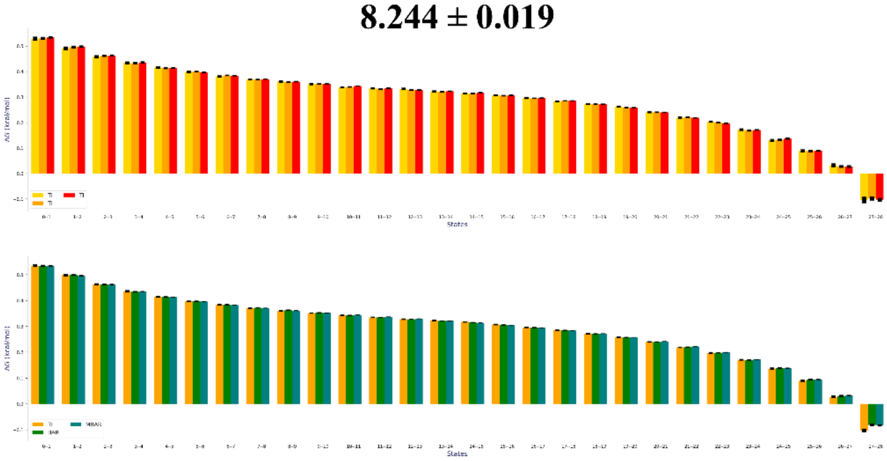

**M06 → M04**

**-8.277 ± 0.017**

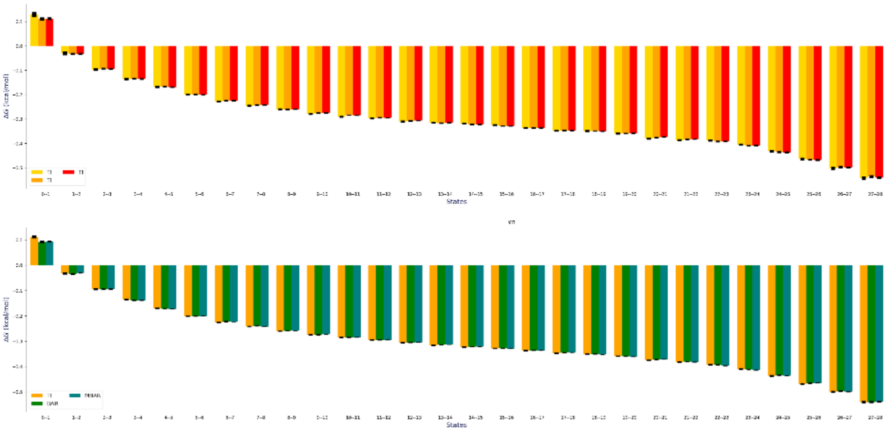

**Figure S6**. TI analysis for the M04↔M06 (forward and backward) transformation in aqueous solution (values in kcal·mol$^{-1}$). Representation of (top) the free energy change determined from the last 6ns of each λ divided in steps of 2 ns (each in yellow, orange and red), and (bottom) correlation between the TI estimator used in the calculations and the BAR and MBAR estimators.

## M04 → M06

**7.897 ± 0.029**

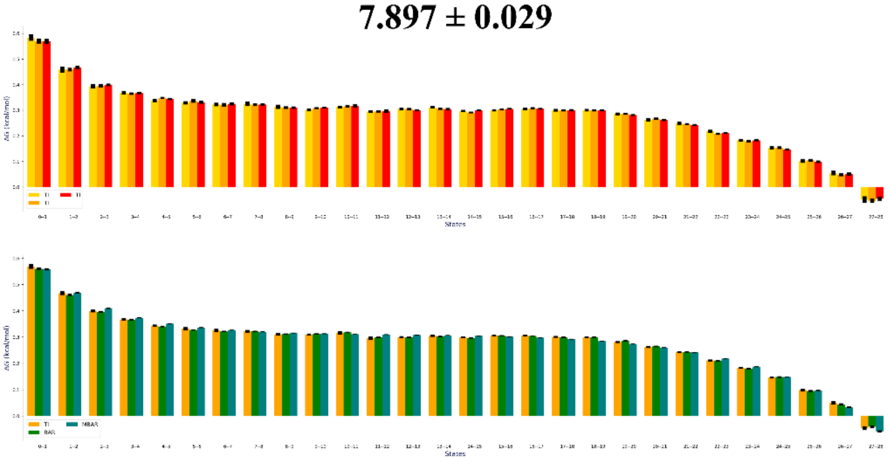


## M06 → M04

**-7.835 ± 0.028**

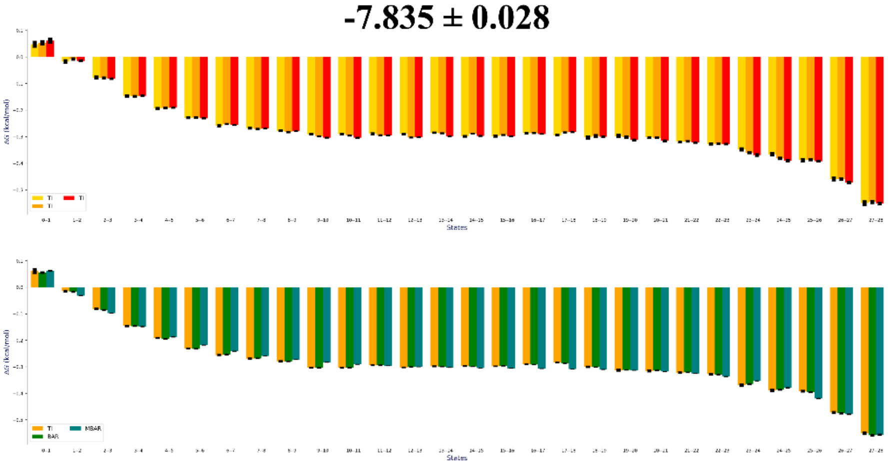


**Figure S7**. TI analysis for the M04↔M06 (forward and backward) transformation in the ligand-protein complex (values in kcal·mol$^{-1}$). Representation of (top) the free energy change determined from the last 6ns of each λ divided in steps of 2 ns (each in yellow, orange and red), and (bottom) correlation between the TI estimator used in the calculations and the BAR and MBAR estimators.

*Principal component analysis*

To explore the conformational flexibility of the structural elements of HA that shape the putative binding pocket for M090 in the wild type and $E74_2 \rightarrow D$ mutated variant, Principal Component Analysis (PCA) was used to examine the structural plasticity of the apo systems and the M090-bound systems in BM-II. Note that the residue $E74_2$ is located distant from the binding pocket, as noted in the distances from the Cα atom from this latter residue to the Cα atom of both D852 and T3091 (see Table S2).

Computation of the PCA was performed for the main chain atoms (C, Cα, N and O atoms) of L1-BS, L2-BS and H-BS. To this end, the trajectories of each system were properly aligned to the α-helix backbone of an average structure computed with the last 100 ns of the H1-WT_II system, which was used as a reference structure. The eigenvalues and eigenvectors were calculated diagonalizing the mass-weighted covariance matrix of the system using the *gmx covar* and *gmx anaeig* modules of the GROMACS 2023.2[S16] package.

**Table S2.** Distance (Å) between the Cα atom of residues $E74_2$ ($D74_2$ in the mutated variant) and $D85_2$ and $T309_1$ in the binding pocket of M090.

| Simulation | $D85_2$ – $E/D74_2$ (Å) | $T309_1$ – $E/D74_2$ (Å) |
|---|---|---|
| Awt | 17.1 ± 0.4 | 24.1 ± 0.6 |
| Amut | 16.7 ± 0.3 | 23.8 ± 0.6 |
| H1wt-II | 17.2 ± 0.4 | 24.2 ± 0.4 |
| H2wt-II | 16.8 ± 0.3 | 24.4 ± 0.5 |
| H3wt-II | 17.2 ± 0.3 | 24.5 ± 0.4 |
| H4wt-II | 17.2 ± 0.4 | 24.3 ± 0.4 |
| H1mut-II | 16.9 ± 0.3 | 24.1 ± 0.5 |
| H2mut-II | 16.8 ± 0.3 | 24.0 ± 0.5 |

**Figure S8**. RMSD profiles determined for the backbone of the residues that define the three binding pockets the MD simulations run for the (top) apo wild type HA and (bottom) its mutated variant.

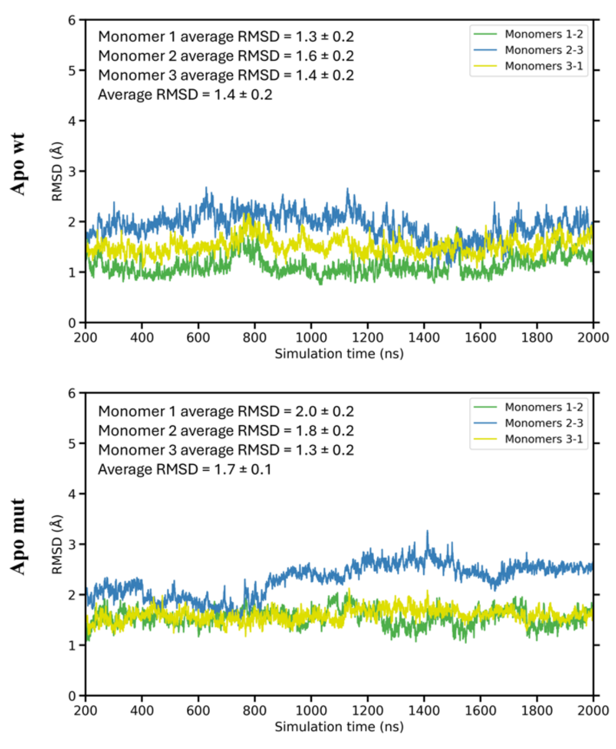

**Figure S9**. RMSD profiles determined for (left) the backbone of the residues that define the three binding pockets and (right) the bound ligand in the two MD simulations (H1mut-II and H2mut-II) run for the complex between M090 (binding mode BM-R) and the mutated variant of HA.

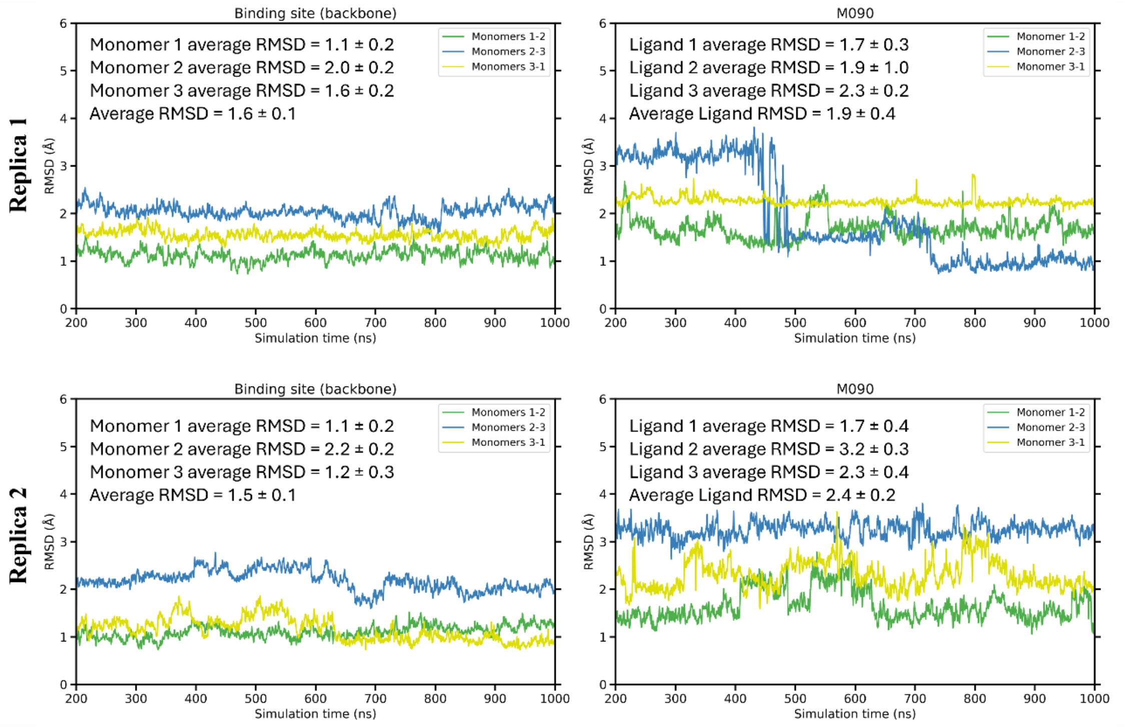

## References


(S1) Waterhouse, A.; Bertoni, M.; Bienert, S.; Studer, G.; Tauriello, G.; Gumienny, R.; Heer, F. T.; de Beer, T. A. P.; Rempfer, C.; Bordoli, L.; Lepore, R.; Schwede, T. SWISS-MODEL: Homology modelling of protein structures and complexes. *Nuc. Acids Res.* **2018**, *46*, W296–W303.

(S2) Yao, Y.; Kadam, R. U.; Lee, C. D.; Woehl, J. L.; Wu, N. C.; Zhu, X.; Kitamura, S.; Wilson, I. A.; Wolan, D. W. An influenza A hemagglutinin small-molecule fusion inhibitor identified by a new high-throughput fluorescence polarization screen. *Proc. Natl. Acad. Sci. USA* **2020**, *117*, 18431–18438.

(S3) Leiva, R.; Barniol-Xicota, M.; Codony, S.; Ginex, T.; Vanderlinden, E.; Montes, M.; Caffrey, M.; Luque, F. J.; Naesens, L.; Vázquez, S. Aniline-based inhibitors of influenza H1N1 virus acting on hemagglutinin-mediated fusion. *J. Med. Chem.* **2018**, *61*, 98–118.

(S4) Maier, J. A.; Martinez, C.; Kasavajhala, K.; Wickstrom, L.; Hauser, K. E.; Simmerling, C. FF14SB: Improving the accuracy of protein side chain and backbone parameters from FF99SB. *J. Chem. Theory Comput.* **2015**, *11*, 3696–3713.

(S5) Wang, J.; Wolf, R. M.; Caldwell, J. W.; Kollman, P. A.; Case, D. A. Development and testing of a general Amber force field. *J. Comput. Chem.* **2004**, *25*, 1157–1174.

(S6) Wang, J.; Cieplak, P.; Kollman, P. A. How well does a restrained electrostatic potential (RESP) model perform in calculating conformational energies of organic and biological molecules? *J. Comput. Chem.* **2000**, *21*, 1049–1074.

(S7) Gaussian 16, Revision B.01, Frisch, M. J.; Trucks, G. W.; Schlegel, H. B.; Scuseria, G. E.; Robb, M. A.; Cheeseman, J. R.; Scalmani, G.; Barone, V.; Petersson, G. A.; Nakatsuji, H.; Li, X.; Caricato, M.; Marenich, A. V.; Bloino, J.; Janesko, B. G.; Gomperts, R.; Mennucci, B.; Hratchian, H. P.; Ortiz, J. V.; Izmaylov, A. F.; Sonnenberg, J. L.; Williams-Young, D.; Ding, F.; Lipparini, F.; Egidi, F.; Goings, J.; Peng, B.; Petrone, A.; Henderson, T.; Ranasinghe, D.; Zakrzewski, V. G.; Gao, J.; Rega, N.; Zheng, G.; Liang, W.; Hada, M.; Ehara, M.; Toyota, K.; Fukuda, R.; Hasegawa, J.; Ishida, M.; Nakajima, T.; Honda, Y.; Kitao, O.; Nakai, H.; Vreven, T.; Throssell, K.; Montgomery, J. A., Jr.; Peralta, J. E.; Ogliaro, F.; Bearpark, M. J.; Heyd, J. J.; Brothers, E. N.; Kudin, K. N.; Staroverov, V. N.; Keith, T. A.; Kobayashi, R.; Normand, J.; Raghavachari, K.; Rendell, A. P.; Burant, J. C.; Iyengar, S. S.; Tomasi, J.; Cossi, M.; Millam, J. M.; Klene, M.; Adamo, C.; Cammi, R.; Ochterski, J. W.; Martin, R. L.; Morokuma, K.; Farkas, O.; Foresman, J. B.; Fox, D. J. Gaussian, Inc., Wallingford CT, 2016.

(S8) Mark, P.; Nilsson, L. Structure and dynamics of the TIP3P, SPC, and SPC/E water models at 298 K. *J. Phys. Chem. A* **2001**, *105*, 9954–9960.

(S9) Joung, I. S.; Cheatham, T. E. Determination of alkali and halide monovalent ion parameters for use in explicitly solvated biomolecular simulations. *J. Phys. Chem. B* **2008**, *112*, 9020–9041.

(S10) Machado, M. R.; Pantano, S. Split the charge difference in two! A rule of thumb for adding proper amounts of ions in MD simulations. *J. Chem. Theory Comput.* **2020**, *16*, 1367–1372.

(S11) Ryckaert, J.-P.; Ciccotti, G.; Berendsen, H. J. C. Numerical integration of the cartesian equations of motion of a system with constraints: Molecular dynamics of N-alkanes. *J. Comput. Phys.* **1977**, *23*, 327–341.

(S12) Case, D. A.; K. Belfon, K.; Ben-Shalom, I. Y.; Brozell, S. R.; Cerutti, D. S.; Cheatham, T. E., III; Cruzeiro, V. W. D.; Darden, T. A.; Duke, R. E.; Giambasu, G.; Gilson, M. K.; Gohlke, H.; Goetz, A. W.; Harris, R.; Izadi, S.; Izmailov, S. A.; Kasavajhala, K.; Kovalenko, A.; Krasny, R.; Kurtzman, T.; Lee, T. S.; LeGrand, S.; Li, P.; Lin, C.; Liu, J.; Luchko, T.; Luo, R.; Man, V.; Merz, K. M.; Miao, Y.; Mikhailovskii, O.; Monard, G.; Nguyen, H.; Onufriev, A.; Pan, F.; Pantano, S.; Qi, R.; Roe, D. R.;

Roitberg, A.; Sagui, C.; Schott-Verdugo, S.; Shen, J.; Simmerling, C. L.; Skrynnikov, N. R.; Smith, J.; Swails, J.; Walker, R. C.; Wang, J.; Wilson, L.; Wolf, R. M.; Wu, X.; Xiong, Y.; Xue, Y.; York, D. M.; Kollman, P. A. (2020), AMBER 2020, University of California, San Francisco.
(S13) Zhao, X.; Li, R.; Zhou, Y.; Xiao, M.; Ma, C.; Yang, Z.; Zeng, S.; Du, Q.; Yang, C.; Jiang, H.; Hu, Y.; Wang, K.; Mok, C. K. P.; Sun, P.; Dong, J.; Cui, W.; Wang, J.; Tu, Y.; Yang, Z.; Hu, W. Discovery of highly potent pinanamine-based inhibitors against amantadine- and oseltamivir-resistant influenza A viruses. *J. Med. Chem.* **2018**, *61*, 5187–5198.
(S14) Röblitz, S.; Weber, M. Fuzzy Spectral Clustering by PCCA+: Application to Markov State Models and Data Classification. *Adv. Data Anal. Classif.* **2013**, *7*, 147–179.
(S15) Tsai, H.-C.; Tao, Y.; Lee, T.-S.; Merz, K. M. Jr.; York, D. M. Validation of free energy methods in AMBER. *J. Chem. Inf. Model.* **2020**, *60*, 5296–5300.
(S16) Abraham, M. J.; Murtola, T.; Schulz, R.; Páall, S.; Smith, J. C.; Hess, B.; Lindah, E. Gromacs: High Performance Molecular Simulations through Multi-Level Parallelism from Laptops to Supercomputers. *SoftwareX* **2015**, *1–2*, 19–25.
